# Response to Tark et al. (doi: 10.1089/jpm.2018.0626): Variations in Physician Orders for Life-Sustaining Treatment Program across the Nation: Environmental Scan

**DOI:** 10.1089/jpm.2019.0181

**Published:** 2019-09-03

**Authors:** Valerie M. Jimenez, Abby Dotson, Susan W. Tolle

**Affiliations:** ^1^ Center for Ethics in Health Care, Oregon Health and Science University, Portland, Oregon.; ^2^ Emergency Medicine, Oregon Health and Science University, Portland, Oregon.; ^3^ Division of General Internal Medicine and Geriatrics, Center for Ethics in Health Care, Oregon Health and Science University, Portland, Oregon.

Dear Editor:

We disagree with a summary claim in the article published in the *Journal of Palliative Medicine*, 2019, titled “Variations in physician orders for life-sustaining treatment program across the nation: Environmental scan.”^1^ The claim that variations in POLST forms make interstate transfer of POLST orders unlikely is counter to our experience in Oregon. Furthermore, we believe the article does not provide sufficient data on problems with interstate transfer of POLST orders especially between states with well-established POLST programs.

Oregon created the POLST program nearly 3 decades ago, using quality data to guide 13 revisions of Oregon's form.^2^ As the authors note, antibiotic orders were removed from our form when a multistate nursing home study found little difference in antibiotic use, regardless of POLST orders.^3^ Recently, Oregon removed orders regarding artificial hydration and nutrition supported by quality data. We believe POLST forms must continue to be revised based on evidence from robust research.

Although Oregon's POLST program and registry have received hundreds of suggestions for quality improvement, we are not aware of an available Oregon POLST form failing to be respected by health care professionals in a bordering state. Those living on either side of Oregon's borders commonly cross state lines to receive care. It has not mattered that Idaho's POLST form is called POST or that Washington's form is a different color. Oregon, Washington, Idaho, and California all have Section A (attempt cardio pulmonary resuscitation [CPR] and do-not-resuscitate [DNR]) and Section B (medical interventions) and although worded slightly differently, it is clear if the patient wants CPR and full treatment, wants to return to the hospital for medical treatments, or wants their care to focus on comfort.^4^ If a nonconforming state did not have core POLST form elements, it is likely that the form would cause confusion. We are not aware of patients attempting to use forms in Oregon from nonconforming states, thus cannot comment on the degree to which they would be honored.

We acknowledge that Oregon's experience may be unique because Oregon, Washington, Idaho, and California all have well-developed POLST programs. Oregon also has reciprocity laws related to advance care planning documents that support respect for medical orders from health care professionals in other states.^4^ Of course, if a state does not have a well-developed program, Emergency Medical Services (EMS) may not have a protocol to honor POLST. In this situation, POLST orders will not be honored by EMS regardless of which state the orders originated.

We recommend that changes to POLST forms and procedures be based on evidence. Currently, there is insufficient data on problems with interstate transfer of POLST orders to warrant changes to POLST forms or procedures. Border-sharing mature programs can serve as a model to other states to ensure POLST orders across state lines are respected. Assuring the wishes of those with advanced illness or frailty are carefully elicited, recorded, and honored requires support of state laws and regulations, extensive ongoing statewide education, continuing quality assurance, and systems that support the respect for patient wishes no matter where they reside or travel.
